# Loss of social independence in patients with neurofibromatosis type 2: a follow-up study using a national registry in Japan

**DOI:** 10.1265/ehpm.22-00222

**Published:** 2023-08-19

**Authors:** Hiroto Okoshi, Takashi Yamauchi, Machi Suka, Hiroyuki Yanagisawa, Masazumi Fujii, Chikako Nishigori

**Affiliations:** 1 Department of Public Health and Environmental Medicine, The Jikei University School of Medicine, Tokyo, Japan; 2 The Jikei University School of Medicine, Tokyo, Japan; 3 Department of Neurosurgery, Fukushima Medical University, Fukushima, Japan; 4 Graduate School of Medicine, Kobe University, Kobe, Japan

**Keywords:** Neurofibromatosis type 2, Social independence, Registry, Follow-up study, Neurological symptoms

## Abstract

**Background:**

For patients with neurofibromatosis type 2 (NF2), maintaining an independent state of living is important. The present study aimed to examine the loss of social independence (i.e., a status that patients can work and go to school) and its contributing factors in patients with NF2 using data from a national registry in Japan.

**Methods:**

This longitudinal study used a registry database containing information on patients with NF2 who had submitted initial claims to receive medical expense subsidies between 2004 and 2010. Patients with “employed,” “studying,” and “housekeeping” categories were classified as “socially independent.” Patients who were socially independent at baseline were followed-up for up to nine years. The primary outcome of the present study was the loss of social independence during the follow-up period, which was defined as the change in status from being socially independent to socially dependent. First, we examined longitudinal associations between demographic variables and neurological symptoms at baseline and the loss of social independence. Second, we examined whether the occurrence of neurological symptoms is associated with a loss of social independence in patients.

**Results:**

A total of 156 patients were included in the present study. During the follow-up period, 37 (23.7%) patients experienced a loss of social independence. In the first analysis, the multivariate logistic regression model showed that the loss of social independence was significantly more frequent among patients with spinal dysfunction than among patients without. In the second analysis, logistic regression analyses showed that neurological symptoms, including bilateral hearing loss, facial nerve palsy, cerebellar dysfunction, decreased facial sensation, speech dysfunction (dysphagia/dysarthria and aphasia), double vision, blindness, hemiparesis, and seizures, were significantly associated with loss of social independence.

**Conclusions:**

The occurrence of various neurological symptoms of NF2 can hinder social independence in the long term. Medical service providers need to observe patients while considering the risks, and provide appropriate support to address neurological symptoms that can restrict social independence, as this will lead to maintaining social engagement.

## Introduction

Neurofibromatosis type 2 (NF2) is an autosomal dominant neurocutaneous disorder that results in multiple neoplasia syndromes, leading to the development of schwannomas, meningiomas, ependymomas, and other tumors. It is a rare disease caused by a mutation in the NF2 tumor suppressor gene located in chromosome 22q12 and has an estimated birth incidence of about one in 25,000 [1, 2]. Although NF2 is an autosomal dominant genetic disease, more than half of the patients show no positive family history [1, 3]. Bilateral vestibular nerve schwannomas are distinctive features of NF2, although other forms of schwannomas (i.e., cranial, spinal, and peripheral nerve schwannomas), as well as meningiomas, ependymomas, astrocytomas, and rarely, neurofibromas, may also develop [1]. Patients show various symptoms, including vertigo and loss of balance, with hearing loss being the major symptom [4]. Although surgical and radiation therapies are offered as treatment options, there is no established treatment for NF2. Further, the use of radiation therapy for patients with NF2 is controversial. Surgical excision may be more difficult after radiosurgery, and the risk of malignant transformation has been reported [3, 5]. Thus, maintaining an independent state of living is an important aspect of NF2 management.

Various symptoms associated with NF2 may hinder social independence in patients [6, 7]. However, the association between the occurrence of neurological symptoms and loss of social independence has not been fully elucidated.

In Western countries, several epidemiological studies have analyzed the characteristics of patients with NF2 using national registries [8, 9], including an online patient-entered database [10]. However, these database- and registry-based follow-up studies have not addressed the status of social independence in this specific population.

In Japan, several studies have examined the characteristics and clinical course of patients with NF2 [11–13], who submitted claims to receive medical expense subsidies, using a national registry of the Ministry of Health, Labour and Welfare (MHLW) [11, 12]. While these studies assessed the clinical course of the disease, including mortality and risk factors for progressive disability, social independence status as a factor was not specifically addressed.

Our previous cross-sectional study analyzing the social independence status of patients with NF2 using the MHLW database revealed that neurological symptoms, such as hearing loss, blindness, hemiparesis, and seizures, were associated with lower social independence [7]. However, the longitudinal associations between neurological symptoms and social independence were not determined due to the cross-sectional design of our study. Therefore, in the present study, we conducted a follow-up evaluation using the MHLW database to examine 1) the factors (i.e., age, sex, or neurological symptoms at baseline) predicting the loss of social independence and 2) whether the occurrence of neurological symptoms is associated with the loss of independence in patients with NF2. Understanding the associated factors is important for promoting social engagement in affected patients.

## Methods

### Data source and study population

The Japanese government has been promoting measures against rare intractable diseases, referred to as *nanbyou*, as described in detail in a previous study [14]. Patients who meet certain severity grades of these diseases are eligible for medical expense subsidies. To receive these subsidies, applicants must submit claims to the prefectural government. Applicants also have to submit a renewal application annually if they want to keep receiving the subsidy. Medical certificates required to receive these subsidies are documented for all patients. The Intractable/Rare Disease Control Division of the MHLW provides access to its database for research purposes. All personally identifiable information, such as names and addresses, is removed, and each patient is coded by a unique digit identification (ID) number. We designed a longitudinal study using datasets such that these initial and annual renewal datasets were connected.

NF2 is an intractable disease designated by MHLW for which medical expense subsidies are available to eligible patients. This longitudinal study used a fully anonymized registry database provided by MHLW comprising information on patients with NF2 in Japan from 2004 to 2010.

Ethical approval was not required for the present study, in accordance with the “Ethical Guidelines for Medical and Health Research Involving Human Subjects” set forth by the Japanese government. The present study used a fully anonymous database provided by the MHLW, which contained no personally identifiable information.

In all cases, the diagnosis of NF2 was made by physicians according to the criteria of the National Institutes of Health [15]. In Japan, patients with at least one of the following neurological symptoms are eligible for medical expense subsidies: hearing loss, facial nerve palsy, cerebellar dysfunction, decreased facial sensation, dysphagia/dysarthria, aphasia, double vision, blindness, hemiparesis, memory loss, seizures, and spinal dysfunction [16]. As described in our previous study, speech dysfunction included dysphagia or dysarthria and aphasia [7]. The database was used to collect data including information on sex, age at baseline, family history of NF2, activities of daily living (living normally, living with disability, partial care, and complete care), social independence status, and neurological symptoms.

### Loss of social independence

The item on social activity in the database consists of the following categories: “employed,” “studying,” “housekeeping,” “cared for at home,” “cared for at a hospital,” or “cared for at a nursing home”. Thus, social independence in our study was categorized as “employed,” “studying,” or “housekeeping,” and social dependence was categorized as “cared for at home,” “cared for at a hospital,” or “cared for at a nursing home.” Patients under care at home, a hospital, or a nursing home were unable to live alone, without any help. Therefore, they were considered as socially dependent, whereas the others were considered as socially independent. The primary outcome of the present study was the loss of social independence, which was defined as the change in status from being socially independent to socially dependent.

### Eligible participants

Data linkage for annual datasets according to ID numbers was performed for initial claims between 2004 and 2010 and renewal claims between 2005 and 2013. Eligibility criteria for participants were 1) age 6–64 years, 2) no duplicate data, and 3) socially independent status at baseline. The age range of 6–64 years was chosen because children in Japan attend elementary school at 6 years, and adults generally retire at 65 years. Data duplication was assessed based on ID numbers.

**Fig. 1:**
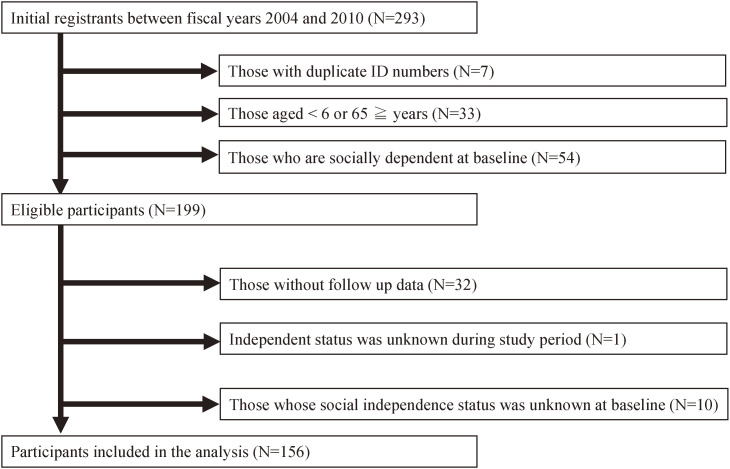
Flowchart for participant selection

Between 2004 and 2010, 293 initial claims were submitted for NF2 medical expense subsidies (Fig. 1). Among them, 94 were deemed ineligible, and 33 were excluded from the analysis due to missing data regarding the social independence status during the follow-up period. Moreover, 10 patients were excluded as their social independence status was unknown at baseline.

### Follow-up

All analyses included in the present study are retrospective analyses. The Intractable/Rare Disease Control Division of the MHLW provides access to its database for research purposes. By connecting the initial and annual renewal datasets, we created the datasets for the present study. Thereafter, patients who were socially independent at the time of registration were followed-up for up to nine years to assess change in status.

### Statistical analyses

Univariate and multivariate analyses using the logistic regression model were performed to examine associations between demographic variables, neurological symptoms, and the loss of social independence, and odds ratios (ORs) with 95% confidence intervals (CIs) were calculated. The outcome variable was a loss of social independence, and the explanatory variables were sex, age (6–24 years, 25–44 years, and 45–64 years), and neurological symptoms (classified as absent/present, or absent/unilateral/bilateral, depending on the type).

Next, to examine whether the occurrence of neurological symptoms is associated with a loss of social independence in patients with NF2, for each neurological symptom, a dataset of participants without the symptoms at baseline was created. They were followed-up for the development of neurological symptoms and the loss of social independence. For example, in a dataset of patients without hearing loss at baseline, we observed only the association between the occurrence of hearing loss and loss of social independence. The symptoms after the loss of social independence were not considered to be associated with this variable. For each dataset, the mean period between the occurrence of neurological symptoms and loss of social independence ranged from 0.1 to 1.0 years; almost all cases of loss of social independence occurred in the same year or the next year as the occurrence of neurological symptoms. Thus, a univariate logistic regression analysis was considered to be suitable and was used to examine the association between the occurrence of a particular neurological symptom and the loss of social independence, calculating odds ratios (ORs) with 95% CIs.

For all analyses, p < 0.05 was considered statistically significant. All analyses were conducted using SAS version 9.4 (SAS Institute, Cary, NC, USA).

## Results

A total of 156 patients (72 males and 84 females) were included in the present study (Table 1). The total number of person-years of observation in the present study was 612 person-years. With respect to the social independence status at baseline, 83 patients were employed, 33 were studying, and 40 were housekeeping. The mean number of patients who submitted initial claims each year was 22.3 (range 19–27). The mean age at baseline was 33.8 (SD 14.6) years, and the median of person-years of observation was 3.0. During the observation period, 37 patients (23.7%) experienced a change in status from socially independent to dependent (19 employed, 10 studying, and 8 housekeeping). The number of participants presenting with NF2 symptoms varied widely by neurological symptoms. Among all patients, 33.3% had unilateral hearing loss and 14.1% had bilateral hearing loss. None had bilateral blindness.

**Table 1:** Characteristics of study participants at baseline

	**n**	**(%)**
**Sex**		
Male	72	46.2
Female	84	53.9
**Age**		
6–24 yrs	50	32.1
25–44 yrs	65	41.7
45–64 yrs	41	26.3
**Family history**		
Present	52	33.3
Absent	74	47.4
Unknown	30	19.2
**Activities of daily living**		
Living normally	65	41.7
Living with disability	73	46.8
Partial care	8	5.1
Complete care	0	0
Unknown	10	6.4
**Hearing loss**		
Bilateral	22	14.1
Unilateral	52	33.3
Absent	82	52.7
**Facial nerve palsy**		
Bilateral	3	1.9
Unilateral	27	17.3
Absent	126	80.8
**Cerebellar dysfunction**		
Present	26	16.7
Absent	130	83.3
**Decreased facial sensation**		
Present	25	16.0
Absent	131	84.0
**Speech dysfunction**		
Present	19	12.2
Absent	137	87.8
**Double vision**		
Present	13	8.3
Absent	143	91.7
**Blindness**		
Bilateral	0	0
Unilateral	3	1.9
Absent	153	98.1
**Hemiparesis**		
Present	8	5.1
Absent	148	94.9
**Memory loss**		
Present	4	2.6
Absent	152	97.4
**Seizures**		
Present	4	2.6
Absent	152	97.4
**Spinal dysfunction**		
Severe	2	1.3
Mild	50	32.1
Absent	104	66.7

### Predictors of loss of social independence

The multiple logistic regression model adjusted for confounding factors revealed that patients with spinal dysfunction (OR 3.0; 95% CI, 1.3–7.2) were significantly more likely to experience loss of social independence during the follow-up period compared to those without (Table 2).

**Table 2:** Logistic regression model of the predictors of loss of social independence at baseline

				**Univariate analysis**			**Multivariate analysis^a^**		

	**All (N)**	**Change in status from socially independent to dependent**		**Odds ratio**	**95% ** **Confidence Interval**	**p value**	**Odds ratio**	**95% ** **Confidence Interval**	**p value**

		**n**	**%**						
**Sex**									
Male	72	19	26.4	1.3	(0.6–2.6)	0.47	1.5	(0.6–3.4)	0.36
Female	84	18	21.4	(ref)			(ref)		
**Age**									
6–24 yrs	50	15	30.0	1.6	(0.7–3.6)	0.30	2.4	(0.9–6.4)	0.09
25–44 yrs	65	14	21.5	(ref)			(ref)		
45–64 yrs	41	8	19.1	0.9	(0.3–2.3)	0.80	1.0	(0.3–3.2)	0.97
**Hearing loss**									
Absent	82	15	18.3	(ref)			(ref)		
Unilateral	52	16	30.8	2.0	(0.9–4.5)	0.10	2.3	(0.9–5.9)	0.10
Bilateral	22	6	27.3	1.7	(0.6–5.0)	0.35	2.4	(0.6–10.3)	0.23
**Facial nerve palsy**									
Absent	126	27	21.4	(ref)			(ref)		
Present	30	10	33.3	1.8	(0.8–4.4)	0.17	1.1	(0.3–3.8)	0.86
**Cerebellar dysfunction**									
Absent	130	26	20.0	(ref)			(ref)		
Present	26	11	42.3	2.9	(1.2–7.1)	0.02	2.8	(0.96–8.4)	0.06
**Decreased facial sensation**									
Absent	131	29	22.1	(ref)			(ref)		
Present	25	8	32.0	1.7	(0.6–4.2)	0.29	0.7	(0.2–3.0)	0.67
**Speech dysfunction**									
Absent	137	30	21.9	(ref)			(ref)		
Present	19	7	36.8	2.1	(0.8–5.7)	0.16	1.1	(0.3–4.1)	0.90
**Double vision**									
Absent	143	33	23.1	(ref)			(ref)		
Present	13	4	30.1	1.5	(0.4–5.1)	0.53	1.5	(0.3–7.2)	0.62
**Blindness**									
Absent	153	36	23.5	(ref)			(ref)		
Present	3	1	33.3	1.6	(0.1–18.3)	0.70	1.2	(0.1–18.9)	0.92
**Hemiparesis**									
Absent	148	35	23.7	(ref)			(ref)		
Present	8	2	25.0	1.1	(0.2–5.6)	0.93	0.6	(0.1–4.0)	0.62
**Memory loss**									
Absent	152	36	23.7	(ref)			(ref)		
Present	4	1	25.0	1.1	(0.1–10.6)	0.95	0.5	(0.03–8.3)	0.64
**Spinal dysfunction**									
Absent	104	18	17.3	(ref)			(ref)		
Present	52	19	36.5	2.8	(1.3–5.9)	0.01	3.0	(1.3–7.2)	0.01

^a^All analyses are adjusted for sex, age (6–24 years, 25–44 years, 45–64 years), and neurological symptoms (classified as absent/present or absent/unilateral/bilateral, depending on the type).

### Associations between the occurrence of neurological symptoms and loss of social independence

As shown in Table 3, the occurrence of neurological symptoms was associated with the loss of social independence. The percentage of participants who experienced a change in status from being socially independent to dependent varied widely by neurological symptoms. Those who had developed bilateral hearing loss (OR 8.6; 95% CI, 2.1–35.5), facial nerve palsy (OR 5.0; 95% CI, 1.9–13.2), cerebellar dysfunction (OR 3.4; 95% CI, 1.2–9.5), decreased facial sensation (OR 3.1; 95% CI, 1.2–8.2), speech dysfunction (OR 6.3; 95% CI, 2.3–17.3), double vision (OR 5.9; 95% CI, 1.6–22.3), blindness (OR 9.3; 95% CI, 1.7–50.1), hemiparesis (OR 6.8; 95% CI, 1.9–24.9), and seizures (OR 13.8; 95% CI 1.5–127.9) during the follow-up period were significantly more likely to experience loss of social independence.

**Table 3:** Cross-tabulation about the associations between the occurrence of neurological symptoms and loss of social independence

	**All (N)**	**Change in status from socially independent to dependent**		**Odds ratio**	**95% CI**	**p Value**
		**n**	**%**			
**Hearing loss (n = 82)**						
Did not occur	48	4	8.3	ref		
Occurred (Unilateral)	18	4	22.2	3.1	(0.7–14.2)	0.14
Occurred (Bilateral)	16	7	43.8	8.6	(2.1–35.5)	<0.01
**Facial nerve palsy (n = 126)**						
Did not occur	103	16	15.5	ref		
Occurred	23	11	47.8	5.0	(1.9–13.2)	<0.01
**Cerebellar dysfunction (n = 130)**						
Did not occur	110	18	16.4	ref		
Occurred	20	8	40.0	3.4	(1.2–9.5)	0.02
**Decreased facial sensation (n = 131)**						
Did not occur	102	13	12.8	ref		
Occurred	29	9	31.0	3.1	(1.2–8.2)	0.02
**Speech dysfunction (n = 137)**						
Did not occur	117	19	16.2	ref		
Occurred	20	11	55.0	6.3	(2.3–17.3)	<0.01
**Double vision (n = 143)**						
Did not occur	133	27	20.3	ref		
Occurred	10	6	60.0	5.9	(1.6–22.3)	0.01
**Blindness (n = 153)**						
Did not occur	146	31	21.2	ref		
Occurred	7	5	71.4	9.3	(1.7–50.1)	0.01
**Hemiparesis (n = 148)**						
Did not occur	137	28	20.4	ref		
Occurred	11	7	63.6	6.8	(1.9–24.9)	0.04
**Memory loss (n = 152)**						
Did not occur	152	36	23.7	ref		
Occurred	0	0		-	-	-
**Seizures (n = 152)**						
Did not occur	147	33	22.5	ref		
Occurred	5	4	80.0	13.8	(1.5–127.9)	0.02
**Spinal dysfunction (n = 104)**						
Did not occur	76	12	15.8	ref		
Occurred	28	6	21.4	1.5	(0.5–4.3)	0.50

## Discussion

The present study examined 1) the predictors of loss of social independence and 2) the associations between the occurrence of neurological symptoms and the loss of social independence using national registry data of patients with NF2 who submitted initial claims to receive medical expense subsidies in Japan. To our knowledge, this is the first study to examine the longitudinal association between neurological symptoms of NF2 and the status of social independence using the MHLW database.

### Predictors of loss of social independence

In patients with spinal dysfunction, a change in status from socially independent to socially dependent occurred likely due to a worsening of the clinical course of the disease. Aboukais et al. reported that spinal tumors are associated with poor prognostic factors in NF2. In their study, patients with spinal tumors were more likely to be diagnosed at a younger age and have more intracranial meningiomas and other intracranial tumors. Moreover, the presence of spinal tumors was associated with nonsense and frameshift mutations [17]. Truncating mutations, such as nonsense or frameshift mutations, reportedly affect mortality and the clinical course of NF2 [8, 18]. Evans et al. reported that patients with such mutations are more likely to have symptoms before 20 years of age and develop at least two symptomatic central nervous systems (CNS) tumors in addition to vestibular schwannomas before 30 years, and have a severe form of the disease [18]. There are at least two recognized subtypes of NF2 with differing clinical courses (i.e., mild vs. severe). Gardner-type NF2 is milder and tends to present later in life (after 25 years), and bilateral vestibular schwannomas are often the only presenting feature. This subtype is generally associated with survival beyond 50 years. Wishart-type NF2 is more severe and has an earlier onset (before 25 years), with multiple (and rapidly progressive) CNS tumors presenting first rather than vestibular schwannomas. Patients with this type of NF2 require repeated surgical interventions, and many often do not survive beyond 50 years of age [19, 20]. The presence of spinal tumors is associated with truncating mutations, which may be associated with Wishart-type NF2.

### Associations between the occurrence of neurological symptoms and the loss of social independence

Patients who had developed bilateral hearing loss, facial nerve palsy, cerebellar dysfunction, decreased facial sensation, speech dysfunction, double vision, blindness, hemiparesis, and seizures during the follow-up period were significantly more likely to experience loss of social independence. These neurological symptoms may be associated with more severe NF2 (i.e., Wishart type).

In those with these neurological symptoms, loss of social independence was likely due to their symptoms and worsened clinical course. Various NF2 symptoms can affect patients’ daily lives and social engagement levels. Lin et al. reported that hearing difficulties are significantly associated with accidental injuries, specifically those related to work or leisure [21]. Accidental injuries may also contribute to future employment and educational difficulties. Liu et al. also reported that hearing loss was associated with dementia, specifically in patients aged 45–64 years [22], leading to further employment difficulties. Other neurological symptoms, such as facial nerve palsy, can also hinder employment and education.

Only spinal dysfunction was found to be significantly associated with loss of independence (Table 2), although many neurological symptoms were found to have a significant association (Table 3). Two reasons for this inconsistency are considered. First, the methods employed to determine significance were different. Table 2 shows the risk factors predicting the loss of social independence at baseline. On the other hand, Table 3 shows the temporality association between the occurrence of neurological symptoms and loss of social independence more direct than in Table 2. Second, the difference in analysis method between Table 2 and Table 3; Table 2 primarily shows the results of multivariate analysis, while Table 3 shows those of univariate analysis.

Although spinal dysfunction was significantly associated with loss of social independence in Table 2, the association was not significant in the univariate logistic analysis in Table 3. This may be due to the low prevalence of severe spinal dysfunction during the follow-up period and the small sample size of the present study, as 52 patients with spinal dysfunction at registration were excluded from the analysis.

Although NF2 is an intractable disease without an established treatment, some measures may help patients continue to lead a socially independent life. For example, hearing loss is the most common symptom of NF2, and Kim et al. recommended annual MRI scans and audiometric examinations, since maintaining hearing in at least one ear is the most important factor in determining optimal intervention time [23]. Furthermore, a previous study reported that hearing loss is associated with dementia. Maintaining hearing ability at a stable level could also contribute to the prevention of dementia [22]. Therefore, we believe improving the social environment and medical service systems is important for patients to easily access medical facilities constantly.

Tan et al. reported that cochlear implantation is effective in auditory rehabilitation and should be considered primarily for patients with NF2 with intact cochlear nerves [24]. Sanna et al. reported that auditory brainstem implantation could provide good communication support for those without intact cochlear nerves, specifically combined with lip reading [25]. A study found that bevacizumab, a vascular endothelial growth factor-neutralizing antibody, is effective for tumor shrinkage and subsequent hearing improvement in these patients [26]. Not all patients have access to auditory brainstem implantation or bevacizumab, as national health insurance does not cover these therapies. In Japan, a randomized controlled trial designed to assess the efficacy and safety of bevacizumab in patients with NF2 with vestibular schwannomas began in October 2019 and is currently ongoing [27], aimed at official approval of the Japanese government and the health insurance service coverage. Further investigations are required to develop effective therapies and support systems for hearing loss and other neurological symptoms.

Finally, patients with rare intractable diseases like NF2 may experience mental health problems. Indeed, in their study, Wang et al. reported that neurofibromatosis (NF1, NF2, or Schwannomatosis) was associated with symptoms of depression and anxiety, as well as higher levels of stress [28]. Meanwhile, economic problems may also affect patients’ mental health. Fukuda et al. reported that lower income and unemployment were associated with poor mental states such as distress or depression [29]. Patients who lose their social independence may lose their income, which can affect their mental health. Therefore, as Wang et al. discussed, we also think that physicians, caregivers, and patients should be aware of the patient’s mental state and provide appropriate support when needed [28]. Moreover, the item regarding patients’ mental states (anxiety or depression) has been added to medical certificates for neurofibromatosis patients since 2015 in Japan. Thus, future work should evaluate the mental health of patients using this data.

### Strengths and limitations

The present study followed NF2 patients for up to nine years to examine change in status from socially independent to dependent using national registry data. The registry used in the present study included all patients with NF2 who had submitted medical certificates filled out by physicians to receive medical expense subsidies in Japan. However, there are some limitations. First, the sample size was not significantly large due to the exclusion of ineligible patients. Moreover, differences in study design and sample size may explain why our findings were not completely consistent with our previous cross-sectional study [7], regarding neurological symptoms associated with social dependence. Second, the MHLW registry database did not include patients with subclinical NF2 who did not seek healthcare services or request medical expense subsidies, potentially leading to selection bias. The database also lacked information on medical history; thus, the change in status from socially independent to dependent might have been due to other underlying disorders. Moreover, the data used in the study did not include detailed information on whether the patients’ physical condition had improved and medical treatment was no longer necessary, whether patients had moved to another prefecture, or died. Third, multivariate analysis considering the follow-up period was not performed in the analysis of associations between the occurrence of neurological symptoms and loss of social independence because (1) for each neurological symptom, a dataset of participants without the symptom at baseline was created and (2) the sample size of each dataset used in the analysis was small. Fourth, we analyzed data obtained from 2004 to 2013. Information about patients registered from 2015 to 2022 was not included because the law for intractable disease was established in Japan in 2015. Moreover, data on social independence were not included in the medical certificates from 2015 to 2022. Fifth, we did not analyze the loss of social independence according to disease severity because it was not included in the medical certificate used in the present study. Finally, epidemiological data from patients with NF2 admitted to medical facilities in Japan are currently unavailable. Thus, we did not compare clinical databases with the MHLW registry database.

## Conclusions

This follow-up study examined the loss of social independence in patients with NF2 in Japan using a national registry. Patients with spinal dysfunction were at higher risk for being socially dependent. Those who had developed neurological symptoms such as bilateral hearing loss, facial nerve palsy, cerebellar dysfunction, decreased facial sensation, speech dysfunction, double vision, blindness, hemiparesis, and seizures were significantly more likely to experience loss of social independence. The occurrence of these neurological symptoms can hinder patients’ social independence in the long term. In addition, medical service providers need to observe patients while considering this risk and provide appropriate support to those developing these neurological symptoms, as they may help patients with NF2 maintain social engagement.

## Abbreviations

MHLW: Ministry of Health, Labour and Welfare
NF2: Neurofibromatosis type 2

## Declarations

### Availability of data and materials

The datasets analyzed in the current study are not publicly available because the national registry of NF2 was provided specifically for the present study by the MHLW.
